# Uptake of Pharmaceutical Pollutants and Their Metabolites from Soil Fertilized with Manure to Parsley Tissues

**DOI:** 10.3390/molecules27144378

**Published:** 2022-07-08

**Authors:** Klaudia Stando, Ewa Korzeniewska, Ewa Felis, Monika Harnisz, Sylwia Bajkacz

**Affiliations:** 1 Department of Inorganic, Analytical Chemistry and Electrochemistry, Faculty of Chemistry, Silesian University of Technology, B. Krzywoustego 6 Str., 44-100 Gliwice, Poland; 2 Department of Engineering of Water Protection and Environmental Microbiology, Faculty of Geoengineering, University of Warmia and Mazury in Olsztyn, Prawocheńskiego 1 Str., 10-720 Olsztyn, Poland; ewakmikr@uwm.edu.pl (E.K.); monikah@uwm.edu.pl (M.H.); 3 Centre for Biotechnology, Silesian University of Technology, B. Krzywoustego 8 Str., 44-100 Gliwice, Poland; ewa.felis@polsl.pl; 4 Environmental Biotechnology Department, Faculty of Power and Environmental Engineering, Silesian University of Technology, Akademicka 2 Str., 44-100 Gliwice, Poland

**Keywords:** pharmaceuticals, parsley, plant metabolism, plant uptake, transformation products, LC-MS/MS analysis

## Abstract

Manure is a major source of soil and plant contamination with veterinary drugs residues. The aim of this study was to evaluate the uptake of 14 veterinary pharmaceuticals by parsley from soil fertilized with manure. Pharmaceutical content was determined in roots and leaves. Liquid chromatography coupled with tandem mass spectrometry was used for targeted analysis. Screening analysis was performed to identify transformation products in the parsley tissues. A solid-liquid extraction procedure was developed combined with solid-phase extraction, providing recoveries of 61.9–97.1% for leaves and 51.7–95.6% for roots. Four analytes were detected in parsley: enrofloxacin, tylosin, sulfamethoxazole, and doxycycline. Enrofloxacin was detected at the highest concentrations (13.4–26.3 ng g^−1^). Doxycycline accumulated mainly in the roots, tylosin in the leaves, and sulfamethoxazole was found in both tissues. 14 transformation products were identified and their distribution were determined. This study provides important data on the uptake and transformation of pharmaceuticals in plant tissues.

## 1. Introduction

Animal manure and liquid manure are commonly used for fertilization and reclamation of agricultural land. Due to their high content of organic matter, nitrogen, phosphorus and micronutrients, they are natural alternatives to nitrogen fertilizers [1]. One of the major concerns of using natural fertilizers is the presence of pharmaceutical residues [2]. Both pharmaceuticals and their metabolites are present in the feces of humans and animals undergoing antibiotic therapy [3]. The use of natural fertilizers in the form of manure results in the introduction and dissemination of pharmaceutical contaminants (FCs) in the environment, and may promote the spread of drug resistance in bacteria [4].

Once released into the environment, depending on their physicochemical properties, FCs can accumulate in the soil, contaminate groundwater, and be uptaken, absorbed or immobilized by plants [5]. FCs can be uptaken with water or nutrients via the root pathway and translocated between tissues [6]. There are three mechanisms of nutrient uptake from the soil via the root pathway: root uptake, mass flow, and diffusion [7]. Accumulation of FCs in plant tissues depends on the cellular structure of the plant’s tissues and the molecular weight, polarity, lyophilicity, and ionic form of these compounds [8].

After absorption of FCs from the soil, they can metabolize in the plant. Transformation products (TPs) of phase I, II, and III metabolism of various FCs have been detected in plant tissues [9]. Tian et al. [9] observed the formation of clarithromycin and sulfadiazine TPs in leaves and roots of lettuce grown under hydroponic conditions in the presence of FCs. Eight clarithromycin metabolites were identified during phase I of plant metabolism and two sulfadiazine metabolites were formed during phase II of metabolism. Other studies have also shown that FCs from the macrolide, tetracycline and sulfonamide groups were metabolized according to phase I or phase II reactions after plant uptake [9].

Absorption and subsequent bioaccumulation and biotransformation of FCs in edible plants carry a risk of transmission to the human gastrointestinal tract [10,11]. Studies on the pharmacokinetics of sulfamethoxazole and tetracycline were conducted using Chinese cabbage (Napa cabbage) and water spinach grown under hydroponic conditions [12]. The study found that the accumulation capacity of the pharmaceutical depends on various physicochemical properties, for example tetracycline had a higher concentration (77–160 µg g^−1^) than sulfamethoxazole (18–38 µg g^−1^). Moreover, both plants showed that the drug accumulated mainly in the roots and to a lesser extent in the green parts, confirming that these compounds were uptaken from the environment. Studies conducted on herbs and grasses treated with penicillin, sulfadiazine and tetracycline confirmed that the roots were most strongly affected by FCs compared to the steams and leaves [13]. Additionally, the presence of FCs in the soil disrupted homeostasis in the plant body, reduced the elemental content of the plant and led to salt stress.

The aim of this study was to evaluate the bioavailability of 14 selected veterinary FCs by parsley plant (*Petroselinum crispum*) from manure-fertilized soil. The conditions for extraction and determination of 14 FCs were developed using high-performance liquid chromatography coupled with tandem mass spectrometer (LC-MS/MS). LC-MS/MS is a superior analytical technique for the determination of trace amounts of FCs in environmental samples [14]. Both the time of flight (TOF) and LTQ-Orbitrap analyzers were preferred due to their high full-scan detection sensitivity, mass accuracy and fast data acquisition. In our research, we used a QTRAP spectrometer, combining the triple quadrupole operating modes with a linear ion trap. This device is suitable for both targeted analyses using the Multiple Response Monitoring Mode (MRM). Additionally QTRAP produces a high amount of fragment ion data which is necessary for retrospective analysis of analyte transformation products. Representatives of FCs from the groups of tetracyclines (tetracycline (TC), oxytetracycline (OTC), doxycycline (DOX)), sulfonamides (sulfamethoxazole (SMX), sulfadiazine (SFD)), fluoroquinolones (ciprofloxacin (CIP), levofloxacin (LVF), enrofloxacin (ENF)), macrolides (clarithromycin (CLR), tylosin (TYL)), metronidazole (MET), trimethoprim (TRI), vancomycin (VAN), and clindamycin (CLD) were selected based on the World Health Organization (WHO) report [15]. The selected FCs include the most commonly used drugs in human and veterinary medicine, however there are few reports of soil contamination in Poland by FCs. In Poland the most commonly applied FCs in pig, bovine and poultry productions are tetracyclines (TC, DOX, OTC), fluoroquinolones (mainly ENF), sulfonamides (mainly SMX and SFD) combined with TRI and macrolides (mainly TYL) [16,17]. The contamination of agricultural soils in northern Poland with residues of selected FCs was also assessed. Selected FCs were present in 21 agricultural soil samples out of 39 tested, and the concentrations of SMX, MET, TRI, TYL, ENF ranged from 3.6 to 57 μg·kg^−1^ [18].

A field experiment was conducted in which two types of animal manure (poultry or cattle) were introduced into the soil, in which parsley was sown and grown. The control sample was unfertilized soil, free from FCs’ contamination. The experiment was conducted over a four-month growing period. Bioaccumulation of FCs was evaluated by determining their concentrations in parsley leaves and roots collected after the plant vegetation period (targeted analysis). Additionally, FCs’ transformation products present in parsley leaves and roots were identified using semi-untargeted and untargeted analysis.

## 2. Materials and Methods

### 2.1. Chemicals

Analytical standards TC, OTC, DOX, ENF, LVF, CIP, TYL, TRI, MET, CLR, CLD, SMX, SFD, and VAN were purchased from Sigma-Aldrich (St. Louis, MO, USA). Hypergrade acetonitrile (ACN), methanol (MeOH) and water were purchased from Merck (Darmstadt, Germany). Analytical-grade formic acid (FA), hydrochloric acid (HCl), acetic acid (AcA), sodium hydroxide (NaOH), ethylenediaminetetraacetic acid (EDTA), 25% ammonium hydroxide solution, MeOH, ethanol, ACN and chloroform were purchased from CHEMMPUR (Piekary Śląskie, Poland). Analytical-grade phosphate dibasic dehydrate (>98%) and citrinic acid monohydrate (>98%) were purchased from Sigma-Aldrich.

OASIS HLB (500 mg, 6 mL), OASIS WCX (60 mg, 3 mL), and OASIS MAX (150 mg, 6 mL) cartridges were purchased from Waters (Eschborn, Germany). BAKERBOND^TM^ Octadecyl (C18) (500 mg, 6 mL) cartridges were purchased from BAKERBOND^®^ (J.T. Baker, Philipsburg, PA, USA).

### 2.2. Preparation of Standard Solutions

The standard stock solutions of all FCs were prepared in 1.0 mg·mL^−1^ concentration. TC, OTC, DOX, TYL, TRI, SMX, ENF, LVF, MET, CLR, and CLD were diluted in MeOH. CIP was diluted in 1% FA in MeOH, VAN in MeOH:H_2_O (1:1; *v*/*v*) and SFD in acetone. Calibration solutions of FCs in the range of 1.0 to 1200.0 ng·g^−1^ were prepared in MeOH. At the validation stage, a mixture of working solutions with a defined concentration were added to the parsley root and leaf (blank samples). All the solutions were stored in the freezer at −18 °C and in the dark. The working solutions were stored for a maximum of one week.

### 2.3. Sample Collection

The field experiment was conducted between June and September 2019. Eight experimental plots of 4 m^2^ were prepared. In the poultry manure samples, the FCs’ concentration was relatively low, DOX and CIP were found at the concentrations of 330.3 ± 40.1 ng·g^−1^ and 30.0 ± 2.5 ng·g^−1^ respectively. In the bovine manure and soil, none of the studied drugs were detected. A low amount of selected FCs in manure samples was the basis for additional supplementation of the manure with 4 selected FCs: DOX, ENF, SMX, TYL at a concentration of 50 µg·g^−1^ manure. The selected FCs are the most commonly used in the treatment of cattle and poultry from among the 14 veterinary pharmaceuticals studied in this work. Six plots were treated with manure, three of which were with bovine manure (PMBA–bovine manure-supplemented plots) and three with poultry manure (PMPA–poultry manure-supplemented plots), respectively. Another two control plots were not fertilized with manure (CP). Parsley (*Petroselinum crispum*) was selected as the crop because of its fast growth and high ecological tolerance to anthropogenic pollution. Parsley samples (roots and leaves) were collected using the envelope method from five points of each plot into sterile plastic containers [19]. The samples were transported immediately under darkness and cool conditions to the laboratory, where composite samples were prepared. Parsley root was separated from the leaf and washed with double distilled water, then the samples from univariate plots were combined to form composite samples, homogenized, frozen at −20 °C, and then freeze-dried. Freeze-drying was carried out under 0.035 mbar at −50 °C ALPHA 1–2 LDplus (CHRIST, Osterode am Harz, Germany). Immediately before extraction, the freeze-dried parsley samples were ground to a powder using an electric grinder MK70 dott (ELDOM, Katowice, Poland).

### 2.4. Selection of Conditions for FCs’ Extraction from Plant Tissues

#### 2.4.1. Extraction of FCs from Parsley Leaves

(a) Examination of Solid-Liquid Extraction (SLE) Procedure

5–15 µg mixture of FCs in 3 mL MeOH was added to 0.5 g of ground freeze-dried parsley leaf. The sample was mixed and the solvent was evaporated naturally in the air. SLE was performed using solutions of McIlvain buffer (pH = 4), MeOH, ethanol, ACN, and their mixtures (Table S1). The volume of extractant was 10–20 mL for a single extraction; for double extraction, 2 × 10 mL or 2 × 15 mL of solvent was used. Samples were shaken for 30 min or 2 × 30 min at 750 rpm (single extraction/double extraction) using Vibramax 100 (Heidolph, Schwabach, Germany). For each repetition, the samples were centrifuged for 10 min at 8000 rpm, and the supernatants were combined after double extraction. For liquid chromatography coupled with tandem mass spectrometry (LC-MS/MS) analysis, 1.0 mL of extract was collected.

(b) Examination of Solid-Liquid Extraction Combined with Liquid-Liquid Extraction (SLE-LLE) Procedure

500 ng of FCs’ standard mixture was added to 0.5 g of freeze-dried parsley leaf, which was allowed to stand for 24 h to adsorb the analytes and evaporate the solvent in the air. The extraction was performed using 10 mL of solvent, and the sample was centrifuged at 8000 rpm for 10 min and the supernatant collected and extracted using 5 mL of chloroform. The extracts were evaporated under a stream of nitrogen and dissolved in 1 mL MeOH:0.1% FA in H_2_O (1:1; *v*/*v*).

(c) Examination of Solid-Liquid Extraction Combined with Solid-Phase Extraction (SLE-SPE) Procedure

500 ng of FCs mixture in MeOH was added to 0.5 g of ground freeze-dried parsley leaf, and the solvent was evaporated. The analytes were extracted using a mixture of McIlvaine buffer (pH = 4):ACN (1:1; *v/v*) (single or double extraction). The extracts were adjusted to pH = 3 using concentrated HCl and diluted to 500 mL using distilled water, so that the volume fraction of organic solvent was below 3%.

The utilization of SPE in the purification step of the extracts was studied. Different types of sorbents were examined, including OASIS HLB (500 mg, 6 mL), OASIS MAX (150 mg, 6 mL), BAKERBONDTM Octadecyl (C18) (500 mg, 6 mL) and tandem OASIS WCX (60 mg, 3 mL) as a precolumn. Sorbents were conditioned in the same manner using 6 mL of MeOH, 0.1 M HCl, and distilled water, respectively. Different solvents or mixtures of solvents were used for the elution of analytes: MeOH, 0.1% acetic acid (AcA) in MeOH, 2% FA in ACN/MeOH mixture (8:2 *v*/*v*), and 0.1% NH_3_ in MeOH, (3–10 mL) (SLE-SPE L1–SLE-SPE L6; Table S2). Eluates were evaporated to dryness under a stream of nitrogen. Immediately before LC-MS/MS analysis, the evaporated residue was dissolved in a mixture of MeOH:0.1% FA in H_2_O (1:1; *v*/*v*).

#### 2.4.2. Extraction of FCs from Parsley Root

500 ng of a standard in MeOH was added to 0.5 g of parsley root powder and left in the air for 24 h to evaporate the solvent. SLE was then performed using 10 mL of solvent or mixture of solvent, including MeOH, ACN, 0.2 M NaOH, acetone, McIlvain buffer (pH = 4), and 0.1 M EDTA (SLE-SPE R1: McIlvaine buffer (pH = 4):ACN (1:1; *v*/*v*); SLE-SPE R2: McIlvaine buffer (pH = 4):MeOH (1:1; *v*/*v*); SLE-SPE R3: McIlvaine buffer (pH = 4):MeOH (1:1; *v*/*v*), McIlvaine buffer (pH = 4):ACN (1:1; *v*/*v*); SLE-SPE R4: McIlvaine buffer (pH = 4):ACN (1:1; *v*/*v*), 0.2 M NaOH:acetone (1:1; *v*/*v*); SLE-SPE R5: McIlvaine buffer (pH = 4):MeOH (1:1; *v*/*v*), 0.2 M NaOH:acetone (1:1; *v*/*v*); SLE-SPE R6: ACN: McIlvaine buffer (pH = 4):0.1 M EDTA (2:2:1; *v*/*v*/*v*)). Single extraction was carried out for 30 min by shaking at 750 rpm, and then centrifuged for 10 min at 8000 rpm. In the case of multiple SLEs, the extracts were combined. Samples were diluted to 250 mL using distilled water and adjusted to pH = 3 using FA. SPE was performed using OASIS HLB sorbents (500 mg, 6 mL). Conditioning was performed using 6 mL of MeOH, 0.1 M HCl, and distilled water, respectively. The sorbents were vacuum-dried for 20 min, and the analytes eluted with 6 mL of MeOH and 4 mL of 0.1% AcA in MeOH. The samples were evaporated to dryness under a stream of nitrogen and dissolved in 1 mL of MeOH:0.1% FA in H_2_O (1:1; *v*/*v*).

### 2.5. Sample Preparation

SLE extraction or two-step SLE combined with SPE were used to extract FCs from plant tissues. The best FCs’ extraction parameters were as follows:

#### 2.5.1. Parsley Leaf–Developed Procedure I (Extraction of TC, OTC, DOX, CIP, ENF, LVF, CLD, VAN)

15 mL of McIlvaine buffer (pH = 4):ACN mixture (1:1; *v*/*v*) was added to 0.5 g of freeze-dried parsley leaf and extracted for 30 min. The sample was centrifuged at 8000 rpm for 10 min, the supernatant collected, and a second extraction was performed using the same solvent mixture. The supernatants were combined, diluted to 500 mL using distilled water and adjusted to pH = 3 using HCl. In the case of SPE extraction, OASIS HLB sorbent (500 mg, 6 mL) was used and conditioned with 6 mL of MeOH, 0.1 M HCl, and H_2_O, respectively. The sample solution was passed through the sorbent at a rate of 3 mL·min^−1^ and then the sorbent was vacuum-dried. Elution was performed using 10 mL of 2% FA in MeOH:ACN (2:8; *v*/*v*) and 10 mL of 0.1% NH_3_ in MeOH. The residue was evaporated to dryness under a stream of nitrogen, dissolved in 1 mL of 0.1% FA in H_2_O:MeOH (1:1; *v*/*v*) and centrifuged at 8000 rpm for 5 min. The supernatant was collected and analyzed using LC-MS/MS.

#### 2.5.2. Parsley Leaf–Developed Procedure II (Extraction of MET, SMX, SFD, TRI, TYL, CLR)

0.5 g of freeze-dried leaf sample was extracted twice with 10 mL of MeOH. Each extraction was conducted by shaking the sample at 750 rpm for 30 min, and centrifuged at 8000 rpm for 10 min. The supernatants from the two extractions were combined and evaporated to dryness under a stream of nitrogen. The residue was then dissolved in 1 mL of 0.1% FA in H_2_O:MeOH (1:1; *v*/*v*) and centrifuged at 8000 rpm for 5 min. The clear supernatant was analyzed using LC-MS/MS.

#### 2.5.3. Parsley Root–Developed Procedure (Extraction of 14 FCs)

10 mL of ACN:McIlvaine buffer (pH = 4):0.1 M EDTA mixture (2:2:1; *v*/*v*/*v*) was added to 0.5 g of freeze-dried parsley root and extracted for 30 min at 750 rpm, and centrifuged at 8000 rpm for 10 min. The supernatant was collected and diluted to 250 mL with distilled water and adjusted to pH = 3 using FA. The sample was applied to OASIS HLB sorbent (500 mg, 6 mL), conditioned as in SLE-SPE procedure for leaves (SLE-SPE L1, Section 2.4.1). Elution was performed using 6 mL of MeOH and 4 mL of 0.1% acetic acid in MeOH. The sample was evaporated to dryness under a stream of nitrogen and dissolved in 1 mL of 0.1% FA in H_2_O:MeOH (1:1; *v*/*v*) immediately before analysis.

### 2.6. Instrumental and Analytical Conditions

Dionex UltiMate 3000 HPLC system (Dionex Corporation, Sunnyvale, CA, USA) equipped with: rapid separation pump, autosampler, thermostatted column compartment was used for the analysis of FCs. Dionex Chromeleon TM 6.8 software was used to control the chromatography system. The HPLC system was coupled with an AB Sciex Q-Trap^®^ 4000 mass spectrometer (Applied Biosystems/MDS SCIEX, Foster City, CA, USA). Detailed conditions for LC-ESI-MS/MS determination of 14 FCs were discussed in our previous publication [20]. Briefly, ZORBAX SB-C3 column (150 mm × 3.0 mm i.d., 5 μm, Agilent Technologies, Santa Clara, CA, USA) column was used for chromatographic separation. Elution was performed using a gradient mixture of 0.1% FA in water and ACN. Total analysis time was 10 min, the column temperature was maintained at 30 °C and the injection volume was 5 μL. All FCs were analyzed in positive ionization mode. Each sample was analyzed in multiple reaction monitoring mode (MRM) using the two highest precursor ion/product ion transitions.

### 2.7. Method Validation

The developed SLE-SPE procedure and LC-MS/MS method for determination of 14 FCs in the parsley root and leaf samples was validated. Linearity range, limit of detection (LOD) and quantification (LOQ), recovery, accuracy, precision and matrix effect were studied by analysis of FCs’ standards. Calibration curves were obtained by analyzing the calibration solutions of concentration between 1 ng·g^−1^ and 1200 ng·g^−1^. Quantitative analysis was conducted by calculating the ratios between each analyte’s peak area and the peak area of the calibration curve. Regression equations for each analyte were obtained using the linear regression method. The degree of curve model fit was assessed using the determination coefficient (R^2^). LOQ was determined as the lowest point on the calibration curve, LOD was calculated as 33% LOQ.

The matrix effects (ME) were evaluated by comparing the peak area of FCs diluted in the blank sample (extract of parsley root or leaf) to the peak area of the analytes diluted with pure solvent. Blank samples of parsley root and leaf were previously analyzed to confirm the absence of any significant peak at MRM transitions. The optimized MS parameters (declustering potential, collision energy, collision cell exit potential) for the selected MRM transitions for each compound were given in our previous publication [20]. Recovery was calculated as the ratio of the measured signal analyte area in the sample after extraction (FCs added before extraction) related to the signal area of the analyte in the matrix solution (FCs added after extraction). The recovery was determined at three concentration levels: low-quality control (LQC = 100 ng·g^−1^), middle-quality control (MQC = 400 ng·g^−1^), and high-quality control (HQC = 1000 ng·g^−1^). Precision and accuracy were determined at the same concentration levels. Accuracy was defined as the relative error (RE), precision was determined to form on the coefficient of variation (CV). Analyses were performed in six replications.

### 2.8. Identification of Transformation Products of Selected FCs in Plant Tissues

Two approaches were used to identify TPs in plant tissue samples: screening and untargeted analysis. Environmental samples were analyzed by LC-MS/MS in different modes of linear ion trap. The ion source parameters and ionization mode were the same as for targeted analysis (Section 2.6). Intelligent data acquisition (IDA) mode was used for screening analysis, combining pseudo-monitoring multiple reactions (p-MRM) mode of operation with enhanced product ion scanning (EPI). The development of p-MRM method consisted of collecting literature data on TPs of selected FCs and their characteristic MRM transitions, and then creating a list of TPs in MRM mode. According to IDA criteria, if a compound’s signal intensity exceeded 500 cps and was in the range of 100–1500 Da, then EPI mode was activated and the full mass spectrum recorded. Confirmation was obtained through comparison of the obtained mass spectra of TPs with those available in the literature [20,21]. The use of p-MRM-IDA-EPI reduced the amount of data obtained in the next step.

For untargeted analysis, full data collection mode (EMS) was used in combination with EPI. IDA criteria were the same as for p-MRM-IDA-EPI. After recording mass spectra by EPI-IDA-EMS for the identified compounds in the sample, the focus was on retrospective analysis of spectra that were not recorded in p-MRM-IDA-EPI. Where possible, the data obtained were confirmed using databases or literature.

## 3. Results and Discussion

### 3.1. Development of the Extraction Procedure for the Isolation of FCs from Plant Tissues

#### 3.1.1. Parsley Leaves–Development of SLE Procedure

McIlvain buffer (pH = 4) and organic solvents, including MeOH, ethanol, ACN, and acetone (Table S1), were used to extract FCs from leaf samples [6,10]. Table S3 shows analyte recoveries obtained using SLE for parsley leaves. The shaking time and intensity were chosen based on the stability data of the determined compounds (unpublished material).

Notably, the utilization of only organic solvents provides inefficient extraction of selected FCs from the parsley matrix. The following extraction efficiencies were obtained: 30.1–80.2% for MeOH, 18.8–87.5% for MeOH:ACN (1:1; *v*/*v*), whereas the lowest efficiency was obtained for ACN with only 2.9–63.2%. According to Table S3, it was possible to perform SLE extraction of 7 analytes (SMX, SFD, CLR, MET, TRI, TYL, ENF) using only MeOH, with recoveries above 60%. Performing double extraction with MeOH resulted in higher recoveries of ENF, TYL, TRI, MET, and SFD (above 80%). In the case of SLE of tetracyclines, sulfonamides, and fluoroquinolones from lettuce, carrot, tomato, and walnut leaf samples, a mixture of ACN:acetone (1:1; *v*/*v*, pH = 3) can be used as the extractant [22,23]. According to reports, the procedure of double extraction of SLE using MeOH and 5% FA in MeOH without a sample purification step gives the selected extraction of 59 FCs from samples of 8 edible plant species [24]. The McIlvaine buffer (pH = 4) (SLE L4) was suitable for the extraction of all analytes with a recovery of 31.5% (LVF)–102.8% (VAN). The pH of the extraction buffer is important to improve the solubility of FCs due to their low pKa in the range 1.8–6.6 for all analytes except CLD and CLR [20].

According to the literature, improved extraction of FCs can be achieved by modifying the composition of the McIlvaine buffer solution through addition of an organic solvent [11,25]. In order to recover analytes not extracted by McIlvaine buffer (pH = 4), a mixture with an organic solvent (methanol or ACN) was prepared in a volume ratio of 1:1. McIlvaine buffer (pH = 4) with an organic solvent increased the recovery of fluoroquinolones, sulfonamides and TRI and CLR. After using ACN mixture, higher recoveries were obtained for tetracyclines (TCs: 71.3–91.6%) and sulfonamides (SAs: 76.8–95.9%), compared to MeOH (TCs: 64.2–79.6%, SAs: 66.2–90.4%). It was found that the presence of McIlvain buffer allowed the extraction of analytes with high polarity, and the addition of ACN allowed the extraction of more non-polar compounds [25,26,27]. Therefore, a mixture of McIlvain buffer (pH = 4):ACN was used in further experiments. The use of double extraction with McIlvain buffer (pH = 4):ACN mixture further increased the recovery (66.0% (CIP)–96.7% (CLR)) (SLE-SPE L7) compared to single extraction (SLE-SPE L5). In the final procedure described in Section 2.4, the solvent volume was increased from 10 mL to 15 mL due to the high solvent absorption of the freeze dried plant material, which significantly reduced the final extractant volume.

The extraction mixture of 0.2 M NaOH with acetone (SLE-SPE L8) for the extraction of fluoroquinolones (FQs) was examined [26]. The recovery of FQs was below 40.1%, and the supernatant solution was turbid and thick. SLE with aqueous NaOH solution is often used to extract plant proteins due to alkaline conditions being easier to cleave H-bonds that stabilize the protein structure [28]. It is known that the extraction of FCs from parsley leaves using 0.2 M NaOH:acetone mixture promotes coextraction of plant proteins followed by denaturation [29]. Therefore, a suitable procedure suitable for soil matrices, would not translate to plant tissue matrices. A mixture of MeOH:EtOH:H_2_O was also studied, as well as a mixture where water was replaced with McIlvaine buffer (pH = 4) (SLE-SPE L9) [30]. The recoveries for both mixtures were lower than McIlvaine buffer (pH = 4):ACN mixture (1:1; *v*/*v*).

#### 3.1.2. Parsley Leaves–Combining SLE-LLE Procedures

Leaves have a complex organic matrix, consisting of compounds such as lignin, cellulose, proteins, flavonoids, tannins and plant pigments that can affect the efficiency of the extraction process [31]. The main problem with SLE for the preparation of leaf samples is co-extraction from the plant pigment matrix. Reports commonly employ chloroform to extract chlorophyll from plants and its mixtures [32]. Chloroform has low polarity compared to the other organic solvents used, with dipole moments of chloroform (1.04 D), ACN (3.92 D), methanol (1.70 D), and water (1.85 D), hence, it could be used to purify plant extracts containing FCs. Figure S1 compares the obtained results after purification of plant extracts, with chloroform in aqueous (aq.f) and organic (org.f) fractions.

The use of chloroform at the LLE stage for purification of SLE extracts did not improve extraction efficiency. It was observed that the recovery of tetracyclines and CLD in the aqueous fraction increased (37.6–83.43%) compared to pure MeOH. However, 23.0–63.3% of some analytes (LVF, ENF, TYL, TRI, MET, CLR) were also soluble in the organic fraction. The partial solubility of the aforementioned six FCs in chloroform was due to the formation of H-bonds between the analytes and chloroform. According to the Lewis theory, chloroform is an electron pair acceptor, and the -NH_2_, -NHR_2_, -NR_3_, -OH, =O groups present in the drug structure act as an electron pair donor [33]. The combined SLE-LLE method significantly degraded the recovery of SAs and VANs. Therefore, the LLE step was abstained for the purification of the plant matrix.

#### 3.1.3. Parsley Leaves–Combining SLE-SPE Procedures

The use of SPE for the purification of parsley leaf extracts was studied. SPE sorbents of different natures were tested: anion-exchangeable (MAX), cation-exchangeable (WCX), hydrophilic-lipophilic balance (HLB) and silica gel modified with octadecylsilane groups (C18). Sorbent selection consisted of two criteria: effective retention of FCs and removal/retention of matrix components. The obtained results are shown in Figure S2. Previous studies have examined the tandem combination of two different sorbents, where one sorbent was used for retention of matrix constituents and the other for retention of analytes. For example, the tandem combination of cation exchange sorbents (SCX and MCX) with HLB has been successfully applied to extract FCs from plant tissues [34,35]. In our study, we tested the tandem combination of WCX and C18 sorbent (for retention of matrix components) with HLB sorbent (for retention of analytes). The results showed that 10 of 14 analytes were retained on WCX sorbent and the recoveries were in the range of 8.1% (SMX)–49.7% (CLR). C18 sorbent retained 12 of 14 analytes with recoveries ranging from 1.9% (TC)–41.1% (SMX). Due to high analyte loss, WCX and C18 as pre-columns in tandem SPE were not used. The employment of strong anion exchange sorbents (SAX/MAX) and/or HLB for the extraction of FCs from solid samples has been extensively reported [21,32] and promotes the separation of analytes in good recoveries. In our study, we also tested the feasibility of MAX and HLB sorbents. The recovery of FCs with MAX sorbent was in the range of 1.1% (MET)–70.1% (CLR), with recoveries below 35% for 9 of 14 analytes. The best recovery was obtained for the HLB sorbent, where all FCs, except metronidazole, had recovery in the range of 44.4% (CLR)–106.8% (OTC). Therefore, the HLB sorbent was selected for further study.

**Figure 1:**
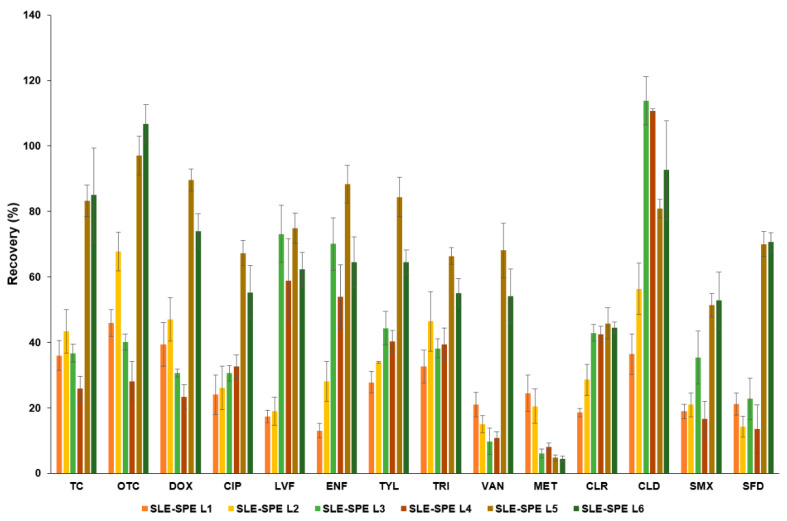
Comparison of the efficiency of FCs’ extraction process following SLE-SPE procedure from parsley leaf.

In the next step, SLE extraction was combined with SPE and parameters such as solvent composition for SLE, sample volume after dilution (so that the volume percentage of organic solvent did not exceed 3%), volume and solvent composition for SPE elution were selected. The selection of solvents for SLE is summarized in Table S3. The comparison of FCs’ extraction efficiency in parsley leaf SLE-SPE procedures (SLE-SPE L1–SLE-SPE L6) is shown in Figure 1, and their parameters are summarized in Table S2. First, one-step (SLE-SPE L1) and two-step (SLE-SPE L2) SLE extraction were performed, followed by SPE extraction under the same conditions. Analyte recoveries for the single extraction were lower (13.0–45.9%) than for the two-step extraction (14.2–67.8%). Commonly, multiple solvent SLE is used for FCs’ extraction from real samples because, in multi-stage extraction, the distribution coefficient is established at each stage, which makes extraction more effective [36,37]. Additionally, the utilization of solvents of different composition at each step as in L2, allows the extraction of FCs with different polarities [6,9,34]. Hence, in SLE-SPE L3 procedure, the volume of elution solution was increased from 10 mL to 20 mL, which increased the recovery of CIP, LVF, ENF, TYL, CLR, and CLD. In another modification (SLE-SPE L4), 0.1% AcA in MeOH and 0.1% NH_3_ in MeOH were used for elution. The application of two-step elution under extreme pH conditions promoted the extraction of a wider group of FCs with different pKa values. Reports have shown that the most common elution of analytes from HLB sorbent after passing the plant matrix extract is carried out under inert conditions–MeOH [6,9], although our study indicated that sequential elution with solvents of different pH gave a higher recovery of FCs.

Finally, we modified the SLE step by increasing the volume of solvent in a single extraction from 10 mL to 15 mL and eliminated the extraction with McIlvain buffer (pH = 4) with MeOH, replacing it with ACN in both extraction steps (SLE-SPE L5, SLE-SPE L6). In addition, 2% FA in a mixture of MeOH:ACN (SLE-SPE L5) and 0.1% AcA in MeOH (SLE-SPE L6) were tested in SPE step as low pH eluent, 0.1% NH_3_ in MeOH was used for the second elution in both cases. Both procedures gave recoveries above 50% for all analytes except MET (4.3–4.7%) and CLR (44.4–45.8%). Application of SLE-SPE L5 and SLE-SPE L6 procedures allowed efficient extraction of 8 FCs: TC, OTC, DOX, CIP, ENF, LVF, VAN, CLD (Figure 1). The SLE-SPE L5 procedure gave better recoveries of the aforementioned compounds compared to SLE-SPE L6, respectively: 68.1% (VAN)–97.1% (OTC), 54.1% (VAN)–106.8% (OTC), hence, it was used as one of the sample preparation procedures for parsley leaves. The efficiency of SLE-SPE L5 extraction was low for the other 6 analytes (SMX, SFD, MET, TRI, TYL, CLR), so it was necessary to use a procedure based on double SLE extraction with MeOH for their separation (SLE L1, without using SPE; recoveries 61.9–89.9%).

#### 3.1.4. Parsley Root–Development of SLE-SPE Procedure

**Figure 2:**
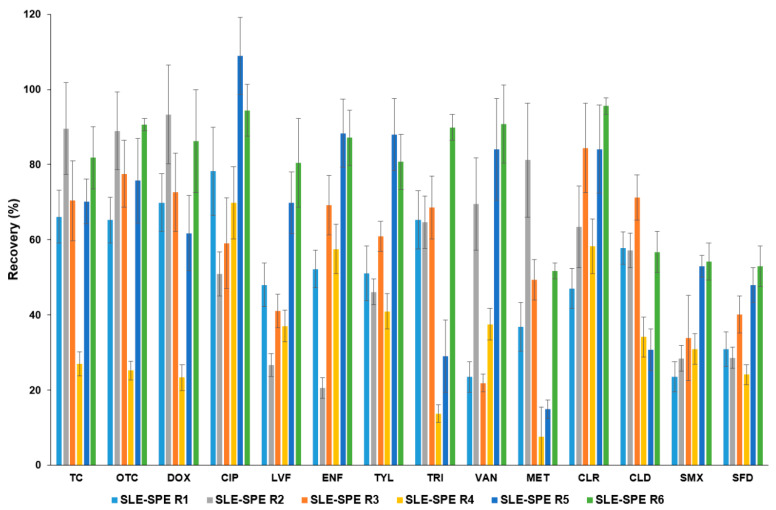
Comparison of efficiencies using SLE-SPE procedures for the separation of analytes from parsley root: SLE-SPE R1: McIlvaine buffer (pH = 4):ACN (1:1; *v*/*v*); SPE-SPE R2: McIlvaine buffer (pH = 4):MeOH (1:1; *v*/*v*); SLE-SPE R3: McIlvaine buffer (pH = 4):MeOH (1:1; *v*/*v*), McIlvaine buffer (pH = 4):ACN (1:1; *v*/*v*); SLE-SPE R4: McIlvaine buffer (pH = 4):ACN (1:1; *v*/*v*), 0.2 M NaOH:acetone (1:1; *v*/*v*); SLE-SPE R5: McIlvaine buffer (pH = 4):MeOH (1:1; *v/v*), 0.2 M NaOH:acetone (1:1; *v/v*); SLE-SPE R6: ACN: McIlvaine buffer (pH = 4):0.1 M EDTA (2:2:1; *v*/*v*/*v*).

SLE-SPE extraction of five selected FCs from parsley root has already been studied by our research team [38]. In the present study, the SLE-SPE procedure was also used to extract 14 selected analytes, and the SLE step was modified. Figure 2 compares the efficiency of FCs’ separation from parsley root using SLE-SPE procedures. In all procedures (SLE-SPE R1, SLE-SPE R6), SPE extraction conditions were the same (Section 2.4.2). The majority of known SLE-SPE procedures have been validated for soil matrices, but less frequently for plant samples. Compared to parsley leaf, parsley root contains much fewer coeluting matrix compounds (essential oils, flavonoids) [39], which affect the extraction efficiency of FCs. Mixtures of McIlvain buffer (pH = 4) with organic solvents (ACN, MeOH) were used for the SLE of soil matrices [25,40]. For extraction of FCs from plant material (roots, leaves, seeds), the same SLE-SPE-based procedures are commonly applied, which differ in analyte recoveries depending on the studied tissue [9,23]. The selection of SLE-SPE extraction conditions from parsley root was similarly performed as for the leaf samples (Section 3.1.1). Performing a single extraction with a mixture of McIlvaine (pH = 4):ACN buffer (SLE-SPE R1) and McIlvaine (pH = 4):MeOH buffer (SLE-SPE R2), gave FCs recoveries of 23.4% (SMX)–78.2% (CIP) and 20.5% (ENF)–93.3% (DOX), respectively. Single SLE-SPE R1 and SLE-SPE R2 extractions were ineffective in the separation of SAs and FQs from the root matrix. Two-step SLE and combination of the mixtures in a single procedure was examined to increase recovery (SLE-SPE R3). In SLE-SPE R3, a significantly higher recovery of CLR (84.4%) and a slight increase in recovery of SMX (33.8%) and SFD (40.1%) were observed. Improved extraction efficiency of fluoroquinolones was examined using extraction under alkaline conditions (SLE-SPE R4, SLE-SPE R5) as the second SLE step, which worked well for soil matrices [26]. The use of a mixture of 0.2 M NaOH with acetone improved the recovery of fluoroquinolones (CIP: 69.8–108.9%, LVF: 37–69.8%, ENF: 57.5–88.3%), but decreased significantly the recovery of TRI, MET, and CLD, hence, this approach was not examined further. Finally, a one-step extraction with a mixture of ACN:McIlvaine buffer (pH = 4):0.1 M EDTA (2:2:1; *v*/*v*/*v*) (SLE-SPE R6) was used, which gave good recoveries for all analytes 51.7–95.6% and the highest repeatability (CV: 2.5–9.6%). Procedures using 0.1 M EDTA in mixtures of different compositions have been reported for soil matrices and manure [25], but have not been applied to the separation of FCs from plant roots.

### 3.2. Method Validation

Tables S4 and S5 present the validation parameters of the developed LC-MS/MS method for parsley root and leaf matrix, respectively. Validation was performed using FCs-free sample extracts prepared according to the procedure described in Section 2.4, enriched with the appropriate amount of analytes. Analyses were performed in six independent replicates. Calibration curves were linear in the range of 1–1200 ng·g^−1^ for all analytes except VAN and TC, for which the range was 5–1200 ng·g^−1^. The coefficient of fit for the curves (R^2^) was in the range of 0.9858–0.9988, indicating a good fit of the curves. The sensitivity of the method was determined by LOD and LOQ, which were 0.3–1.6 ng·g^−1^ and 1.0–5.0 ng·g^−1^, respectively.

Accuracy and precision were determined at three concentration levels (100 ng·g^−1^, 400 ng·g^−1^, 1000 ng·g^−1^). The precision determined as RE (%) was less than 9.60% for the extracts from parsley root and 7.90% from parsley leaf extracts. The precision of the method determined by CV was in the range of 0.40–7.56% for the root matrix and 0.49–11.42% for the parsley leaf matrix. The recoveries determined for the parsley root matrix were in the range of 51.7% (MET)–95.6% (CLR) in HQL samples. In the case of the parsley leaf sample, the recoveries of 6 analytes (SMX, SFD, MET, TRI, TYL, CLR) in double MeOH extraction procedure were in the range of 61.9% (CLR)–89.9% (TYL) in HQL samples. In the parsley leaf SLE-SPE procedure, the recoveries of the remaining 8 analytes (TC, OTC, DOX, CIP, ENF, LVF, VAN, CLD) were in the range of 67.3% (CIP)–97.1% (OTC) in HQL samples. The recoveries obtained were satisfactory and similar to those previously reported for plant matrices [36,41]. The selectivity of the method was obtained by comparing the chromatograms recorded in MRM mode for the analytes after extraction of the plant material and the blank. No interference of matrix components was observed in the chromatograms, with retention times corresponding to the analytes.

The matrix effect was also examined for both extracts from parsley root and leaf samples (Tables S4 and S5). The matrix effect was influenced by both matrix type and sample preparation [42]. It was observed that the matrix effect significantly affected the signal intensity of analytes SFD (signal attenuation, ME = −11.35%) and CLD (signal enhancement, ME = 11.31%) for parsley root matrix. For the other analytes, the matrix effect was negligible (ME < 7.81). Similarly for parsley leaf, where ME was in the range of −6.74–7.96%.

### 3.3. Investigation of FCs Accumulation in Plant Tissues

Four FCs were detected in plant tissue samples collected from plots enriched with poultry manure (PMPA) and cattle manure (BMPA): DOX, TYL, SMX and ENF (Table 1). ENF was detected at the highest concentration, ranging from 23.9–29.3 ng·g^−1^ in parsley leaves, and detected at 13.4–25.3 ng·g^−1^ in parsley roots (Table 1). For example, in a report, three crop species (soybean, corn, and bean) were examined, where root absorption was the main route of ENF uptake by the plant, and its content was in the range of 1.68 ng g^−1^–26.17 µg·g^−1^ [43]. Parsley samples showed slightly higher ENF content in parsley leaves than parsley roots. The different tendency in accumulation was probably related to morphophysiological differences between plant species, in which the type and distribution of tissues and the presence of apoplastic barriers limited the transport of selected ionic forms of pharmaceuticals in the plant [44]. SMX was detected in trace amounts (<6.83 ng·g^−1^) in all parsley samples grown in manure-enriched soil (PMPA, BMPA). According to literature data, SMX is present in the soil environment in anionic form, which causes repulsion from the root cell apoplast and reduces its uptake from the soil [45]. Another reason for low uptake may be the rapid degradation rate of SMX in the soil under environmental conditions [45]. TYL was only detected in parsley leaves, but its concentration was below LOQ (<1.0 ng·g^−1^). In 11 vegetables grown in TYL-enriched soil, 0.2–2.4 ng·g^−1^ was detected, and in most cases the result was below LOQ [3]. According to the literature, TYL in water, soil or manure environment is biodegradable up to 30 days [46]. Our results for parsley samples confirmed the low absorption of TYL by the plant.

In the case of DOX, higher concentrations were detected in the roots (13.62–14.02 ng·g^−1^) than in the green part of the plant (2.06–3.08 ng·g^−1^). DOX in soil is resistant to degradation processes and has a half-life of 533 days [47]. Additionally, it does not show toxic properties to plants and adult earthworms [48]. However, studies conducted by Litskas et al. [49] concurrently confirm that DOX has an inhibitory effect on earthworm reproduction in soil, where the abundance of juvenile organisms was significantly lower in soil enriched with this FC. DOX is mobile in the environment and detectable in soil solutions [48]. It is the least studied pharmaceutical in the whole group of tetracyclines, where studies have shown that DOX was not detected in lettuce [36], while it was present in radish and pak choi [50] when grown in soil supplemented with DOX. The range of concentrations of pharmaceuticals determined in our study (1.0–29.3 ng g^−1^) was comparable to those reported, where 1.6–2.8 ng·g^−1^ ENF, 0.5–1.5 ng·g^−1^, TYL and SMX 0.05–6.8 ng·g^−1^ were detected [10].

Once absorbed by edible plants, FCs are carried back into the food chain, potentially endangering human health. FCs’ residues in edible plants induce antibiotic resistance development and promote the transfer of antibiotic-resistant bacteria to humans [51,52]. In a study conducted in Germany for 1001 food plants for 5 bacterial strains, the drug resistance genes DOX, ENF, SMX and TYL were detected. Relatively high resistance rates *E. faecalis* were also observed for doxycycline (23%) and tylosin (10%) [53]. Another concern about FCs in food is the development of severe allergic reactions. The constant consumption of even low amounts of FCs in food can reduce fertility, be carcinogenic and cause obesity [54].

### 3.4. Identification of Transformation Products of Selected FCs in Plant Tissues

The photolysis of selected FCs is limited to the top layer of soil where light reaches (7–8 mm), and its rate is two orders lower than for the process carried out in water [55,56]. Direct photodegradation is not considered the main pathway for FCs’ degradation in soils. FCs are efficiently transformed and degraded by bacterial strains and fungi present in soils [56,57]. Thus, it was assumed that both TPs formed in the soil and adsorbed by the plant and those formed by FCs’ metabolism may be present in the tissues of harvested plants.

10 ENF TPs and two DOX TPs were detected in plant samples grown in manure-fertilized soils. SMX216 was detected in parsley root samples from PMPA and BMPA plots, and SMX158 was only detected in parsley root from PMPA plot. Table 2 shows the distribution of TPs according to the plot where parsley was grown. Structure identification of TPs was performed in two steps: first, screening analysis using pseudo-MRM mode; then structures were confirmed by recording mass spectra using EMS-IDA-EPI. Table 3 shows the structures of the identified TPs and their characteristic p-MRM transitions. The obtained results were confirmed based on literature data.

According to the obtained results, different tissue distribution of ENF TPs was observed, four ENFs were detected exclusively in leaves (ENF282, ENF376, ENF334, ENF306), four exclusively in roots (ENF330, ENF390, ENF223, ENF291), and two (ENF263, ENF342) in both leaf and root. Two TPs formed by attachment of a hydroxyl group to a quinolone ring (ENF376) or piperazine ring (ENF390) were identified. In ENF390, the ethyl chain at the nitrogen atom on the piperazine ring was additionally oxidized. ENF328 was formed by defluorination of ENF, cleavage of the ethyl group from the piperazine ring, and subsequent oxidation. Hydroxylation and oxidation are typical reactions in biotic transformations of FCs [58]. ENF376 has been described as a ENF metabolite produced by basidiomes [59] and detected in chicken tissues after ENF administration [60]. To date, ENF376, ENF390 and ENF328 have only been identified after ENF photodegradation processes [61,62]. The four ENF TPs were formed by piperazine ring opening. ENF306 was formed by dissociation of the ethyl group from the piperazine ring and opening with loss of C_2_H_4_. For ENF334, loss of C_2_H_4_ from the piperazine ring was also observed, but a carbonyl group was attached at the nitrogen atom instead of an ethyl group. ENF330 was formed by dissociation of fluorine and ethyl group and opening of the piperazine ring followed by oxidation. ENF291 was formed by cleavage of the piperazine ring and loss of the secondary amine nitrogen, followed by oxidation of the methylamine group. Therefore, the transformation of FQs present in soil was mainly by oxidation of the piperazine side chain, although the fluoroquinolone ring remained intact [55]. ENF306, ENF330, and ENF291 are photodegradation products of ENF or its metabolite CIP [62,63], and ENF334 is a metabolite isolated from pharmaceutical slime [64]. Hence, it was possible that these four TPs were formed in the environment and uptaken by the plant. Three TPs were formed by detachment of the main groups that form ENF. ENF342 was formed by dissociation of fluorine from the quinolone ring. Fluorine disconnection occurred by photodegradation [62] and microbial degradation [20]. ENF263 was formed by C-N bond disruption and piperazine ring disconnection. ENF223 was formed by dissociation of the cyclopropyl ring from the ENF263 molecule. According to the literature, ENF263 and ENF223 are formed by biodegradation carried out by microorganisms [58,64]. DOX accumulated mostly in the parsley roots, its two TPs, DOX399 and DOX274, were detected. DOX399 was formed via C-C bond cleavage and dissociation of the amide group from the DOX ring. The transformation pathway leading to the formation of DOX274 from DOX was studied in photodegradation and involved C-C bond breaking, C-N bond breaking and dehydration reactions sequentially [65]. The transformation pathways of DOX under environmental conditions have not been reported in detail. DOX TPs were detected in the photodegradation process [65], but it was disregarded that they were formed by microbial activity or plant metabolism. Two SMX TPs were also detected in parsley roots. SMX158 was formed via S-N bond cleavage and detachment of the isoxazole ring. SMX216 was formed via isoxazole ring opening and dissociation of the -C_2_H_4_ group. Reports have described SMX158 TPs in the degradation of SMX under ionizing radiation, which may suggest that it was formed by SMX oxidation [66] and microorganisms [67]. However, SMX216 was identified as a metabolic product of sulfate-reducing bacteria, hence, it was observed more frequently in oxygen-deficient environments in its free form [68]. As noted, transformation of pharmaceuticals in the environment can occur due to biotic (microbial activity) and abiotic (photolysis) factors [43]. The response of the plant organism to FCs’ transformation products and their tissue distribution depends on the plant species [43,44]. The metabolism of FCs is generally considered to be the detoxification mechanism of the plant; however, there is concern that some metabolites may have the ability to acquire or potentiate bactericidal activity [9,44].

## 4. Conclusions

In this study, efficient methods were developed for the extraction of 14 common FCs’ contaminants from parsley root and leaf samples. The LC-MS/MS technique was used for determination. Depending on the matrix and compound properties, double SLE with MeOH (leaf tissue: TYL, TRI, MET, CLR, SMX, SFD) or combined SLE-SPE (leaf tissue: TC, OTC, DOX, CIP, ENF, LVF, CLD, VAN; root tissue: all FCs) was used. Analyte recoveries from leaf and root tissues were at satisfactory levels (51.7–97.1% for HQL samples), and LOQs ranged from 1–5 ng·g^−1^.

Veterinary drug residues introduced with animal manure to the soil were adsorbed by plants to varying degrees. DOX, TYL, SMX and ENF were detected in plant samples. DOX bioaccumulated mainly in parsley roots, whereas higher concentrations of ENF were detected in parsley leaves. TYL was not detected in plant roots, and concentrations in leaves were below LOQ, indicating its poor bioaccumulation in the plant. SMX was detected in all plants grown in manure-amended soil. Screening analysis identified 14 transformation products of ENF, SMX, and DOX. ENF TPs were formed through hydroxylation, oxidation, and piperazine ring opening reactions. In the case of SMX and DOX, TPs were formed by C-C and S-N bond-breaking.

Due to the widespread practice of zoonotic fertilizer utilization, FCs can be transferred to food crops and subsequently enter the human food chain. Although the concentrations of detected FCs in parsley were at low levels (<29.26 ng·g^−1^), their ability to accumulate and further metabolize in living tissues is of concern. Further studies are needed to determine the extent of the risks from the use of zoonotic fertilizers.

## Figures and Tables

**Table 1:** Concentrations of selected pharmaceuticals in plant tissues.

Concentration (ng·g^−1^ Dry Weight) (SD)						
	PMPA		BMPA		CP	
Tissues	leaf	root	leaf	root	leaf	root
TC	nd	nd	nd	nd	nd	nd
OTC	nd	nd	nd	nd	nd	nd
DOX	2.1 (0.7)	14.0 (0.5)	3.0 (0.1)	13.6 (4.4)	nd	nd
CIP	nd	nd	nd	nd	nd	nd
ENF	23.9 (3.0)	13.4 (0.5)	29.3 (3.9)	25.3 (3.7)	nd	nd
LVF	nd	nd	nd	nd	nd	nd
MET	nd	nd	nd	nd	nd	nd
CLR	nd	nd	nd	nd	nd	nd
CLD	nd	nd	nd	nd	nd	nd
SMX	2.3 (0.1)	<LOQ	2.3 (0.2)	6.8 (2.2)	nd	nd
SFD	nd	nd	nd	nd	nd	nd
TYL	<LOQ	nd	<LOQ	nd	nd	nd
TRI	nd	nd	nd	nd	nd	nd
VAN	nd	nd	nd	nd	nd	nd

PMPA-soil plots supplemented with poultry manure; BMPA-soil plots supplemented with bovine manure; CP-control plots without supplementation; nd–not detected.

**Table 2:** Identified transformation products in parsley leaf and root samples.

Lp.	TPs	PMPA		BMPA		CP	
		Leaf	Root	Leaf	Root	Leaf	Root
1	ENF263	+	+	+	+	−	−
2	ENF330	−	+	−	+	−	−
3	ENF390	−	+	−	+	−	−
4	ENF282	+	−	+	−	−	−
5	ENF342	+	−	−	+	−	−
6	ENF376	+	−	+	−	−	−
7	ENF334	+	−	+	−	−	−
8	ENF306	+	−	+	−	−	−
9	ENF223	−	+	−	+	−	−
10	ENF291	−	+	−	+	−	−
11	DOX274	−	+	−	+	−	−
12	DOX399	−	+	−	+	−	−
13	SMX158	−	+	−	−	−	−
14	SMX216	−	+	−	+	−	−

**Table 3:** Compilation of the determined pharmaceuticals and their identified transformation products.

Analytes	Formula	[M + H]^+^ (m/z)	Fragmentation Ions (m/z)	Structure	Ref.
Pharmaceuticals					
DOX	C_22_H_24_N_2_O_8_	445.2	428.2 154.1	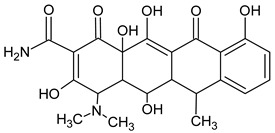	[20]
ENF	C_19_H_22_FN_3_O_3_	360.7	316.2 245.1	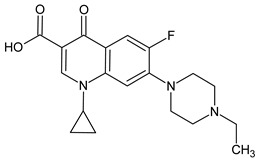	
TYL	C_46_H_77_NO_17_	916.1	174.2 775.5	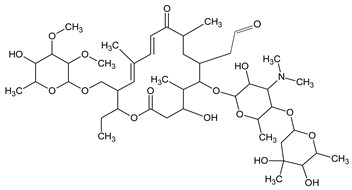	
SMX	C_10_H_11_N_3_O_3_S	253.9	92.0 108.0	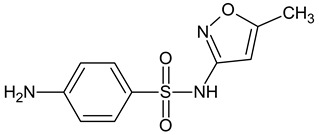	
Transformation products					
ENF263	C_13_H_11_FN_2_O_3_	263.0	245.0 204.0	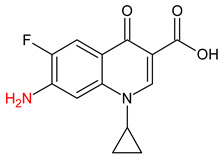	[46,49,69,70,71]
ENF330	C_17_H_19_N_3_O_4_	330.0	312.0 284.0	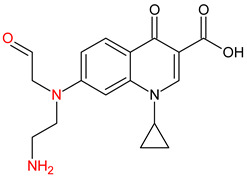	[62]
ENF390	C_19_H_20_FN_3_O_5_	390.0	372.0	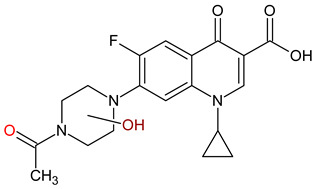	[61]
ENF328	C_17_H_17_N_3_O_4_	328.0	310.0 300.0	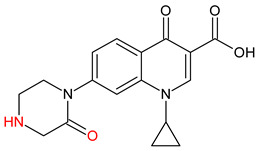	[62]
ENF342	C_19_H_23_N_3_O_3_	342.0	324.0 301.3 297.0	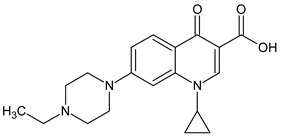	[62]
ENF376	C_19_H_22_FN_3_O_4_	376.1	358.1 340.1	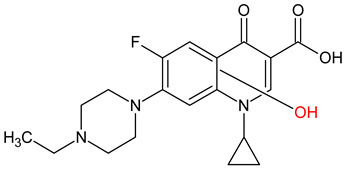	[59,62,72]
ENF334	C_16_H_16_FN_3_O_4_	334.0	316.0 217.0	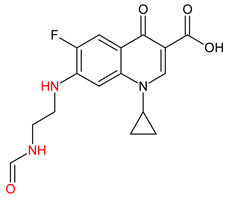	[63,64]
ENF306	C_15_H_16_FN_3_O_3_	306.9	288.0 271.0 145.0	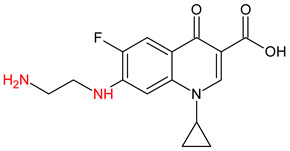	[73,74]
ENF223	C_10_H_7_FN_2_O_3_	223.0	207.0 190.0	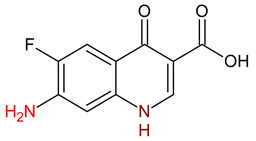	[64]
ENF291	C_14_H_11_FN_2_O_4_	291.0	273.0 245.0 217.0	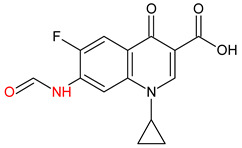	[63]
DOX274	C_16_H_18_O_4_	275.0	275.0 257.0 247.0 229.0	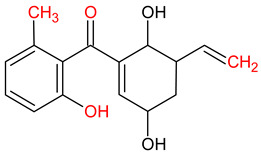	[65], this work
DOX399	C_21_H_21_NO_7_	400.0	400.0 283.0 265.0 211.0	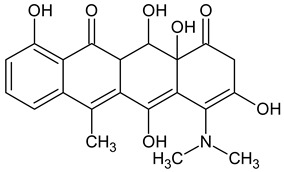	[75], this work
SMX158	C_6_H_7_NO_2_S	158.0	140.0 92.0	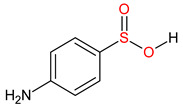	[66]
SMX216	C_7_H_9_N_3_O_3_S	216.0	156.0 202.2	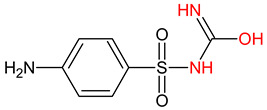	[68]

## Data Availability

All data are included in the article.

## Supplementary Materials

The following supporting information can be downloaded at: https://www.mdpi.com/article/10.3390/molecules27144378/s1, Figure S1: Comparison of the efficiency of analyte extraction following SLE procedure combined with LLE purification stage (matrix: parsley leaves: LLE solvent: chloroform). Figure S2: Comparison of sorbents used for purification of extracts in SLE-SPE procedure of parsley leaf. Eluent composition for MAX, C18, HLB: 0.1% AcA in MeOH (10 mL), 0.1% NH_3_ in MeOH (10 mL), for WCX:MeOH (3 mL), 2% FA in MeOH/ACN (2:8; *v/v*) (3 mL). Table S1: Variable parameters in tested SLE procedures. Table S2: Variable parameters in tested SLE-SPE procedures. Table S3: The application of different solvents to SLE procedure for parsley leaf samples. Table S4: The analytical method parameters and extraction recovery of antibiotics in parsley root samples at three different concentrations (*n* = 6). Table S5: The analytical method parameters and extraction recovery of antibiotics in parsley leaf samples at three different concentrations (*n* = 6).

## Sample Availability

Samples of the compounds are not available from the authors.
