# Supplementary material for: Uptake of Pharmaceutical Pollutants and Their Metabolites from Soil Fertilized with Manure to Parsley Tissues
Source: Molecules. 2022 Jul 8;27(14):4378. doi: 10.3390/molecules27144378 (PMC9317704; doi:10.3390/molecules27144378)
Supplement: Supplementary file 1 [file molecules-27-04378-s001.zip › molecules-1785474-supplementary.pdf]

Supplementary Material

Uptake of Pharmaceutical Pollutants and Their Metabolites from Soil Fertilized with Manure to Parsley Tissues

Klaudia Stando [1,*], Ewa Korzeniewska [2], Ewa Felis [3,4], Monika Harnisz [2] and Sylwia Bajkacz [1,3,*]

[1] Department of Inorganic, Analytical Chemistry and Electrochemistry, Faculty of Chemistry, Silesian University of Technology, B. Krzywoustego 6 Str., 44-100 Gliwice, Poland
[2] Department of Engineering of Water Protection and Environmental Microbiology, Faculty of Geoengineering, University of Warmia and Mazury in Olsztyn, Prawocheńskiego 1 Str., 10-720 Olsztyn, Poland; ewakmikr@uwm.edu.pl (E.K.); monikah@uwm.edu.pl (M.H.)
[3] Centre for Biotechnology, Silesian University of Technology, B. Krzywoustego 8 Str, 44-100 Gliwice, Poland; ewa.felis@polsl.pl
[4] Environmental Biotechnology Department, Faculty of Power and Environmental Engineering, Silesian University of Technology, Akademicka 2 Str., 44-100 Gliwice, Poland
* Correspondence: klaudia.stando@polsl.pl (K.S.); sylwia.bajkacz@polsl.pl (S.B.)

This supporting information file includes additional results and information as described in the text of the main article including:

Figure S1. Comparison of the efficiency of analyte extraction following SLE procedure combined with LLE purification stage (matrix: parsley leaves: LLE solvent: chloroform).

Figure S2. Comparison of sorbents used for purification of extracts in SLE-SPE procedure of parsley leaf. Eluent composition for MAX, C18, HLB: 0.1% AcA in MeOH (10 mL), 0.1% NH_3 in MeOH (10 mL), for WCX: MeOH (3 mL), 2% FA in MeOH/ACN (2:8; v/v) (3 mL).

Table S1. Variable parameters in tested SLE procedures.

Table S2. Variable parameters in tested SLE-SPE procedures.

Table S3. The application of different solvents to SLE procedure for parsley leaf samples.

Table S4. The analytical method parameters and extraction recovery of antibiotics in parsley root samples at three different concentrations (n = 6).

Table S5. The analytical method parameters and extraction recovery of antibiotics in parsley leaf samples at three different concentrations (n = 6).

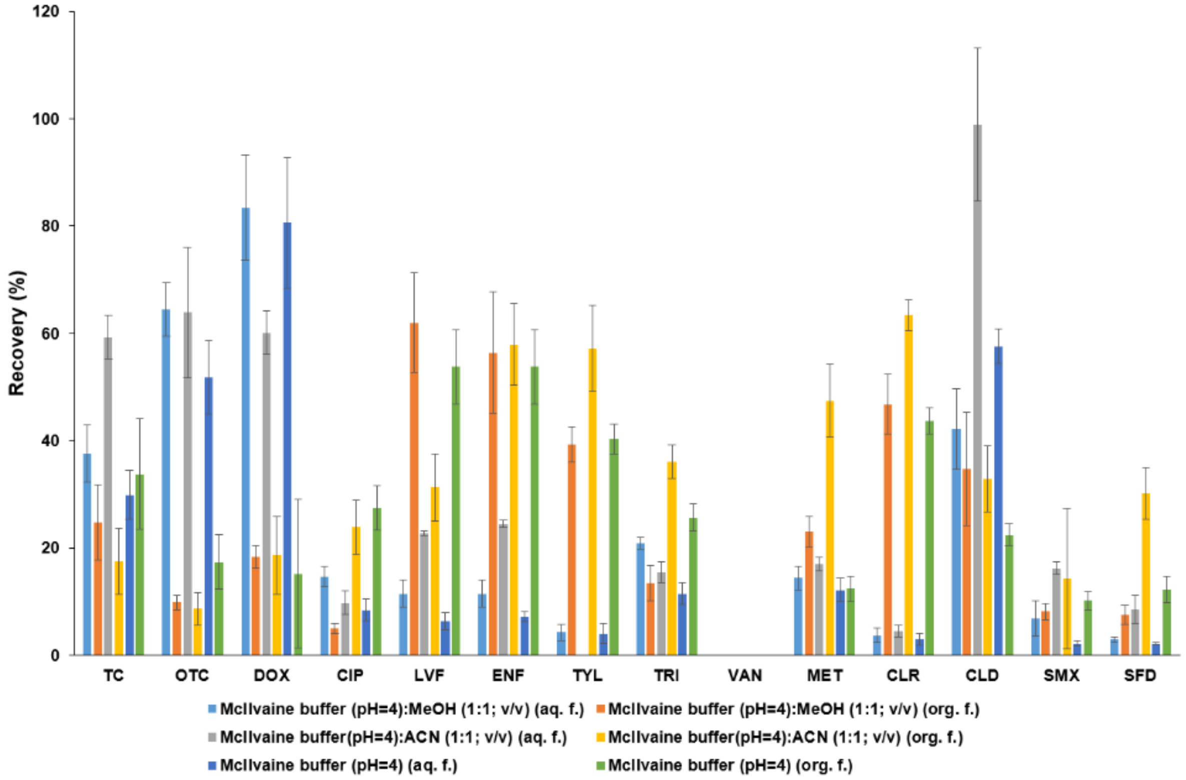

Figure S1. Comparison of the efficiency of analyte extraction following SLE procedure combined with LLE purification stage (matrix: parsley leaves: LLE solvent: chloroform).

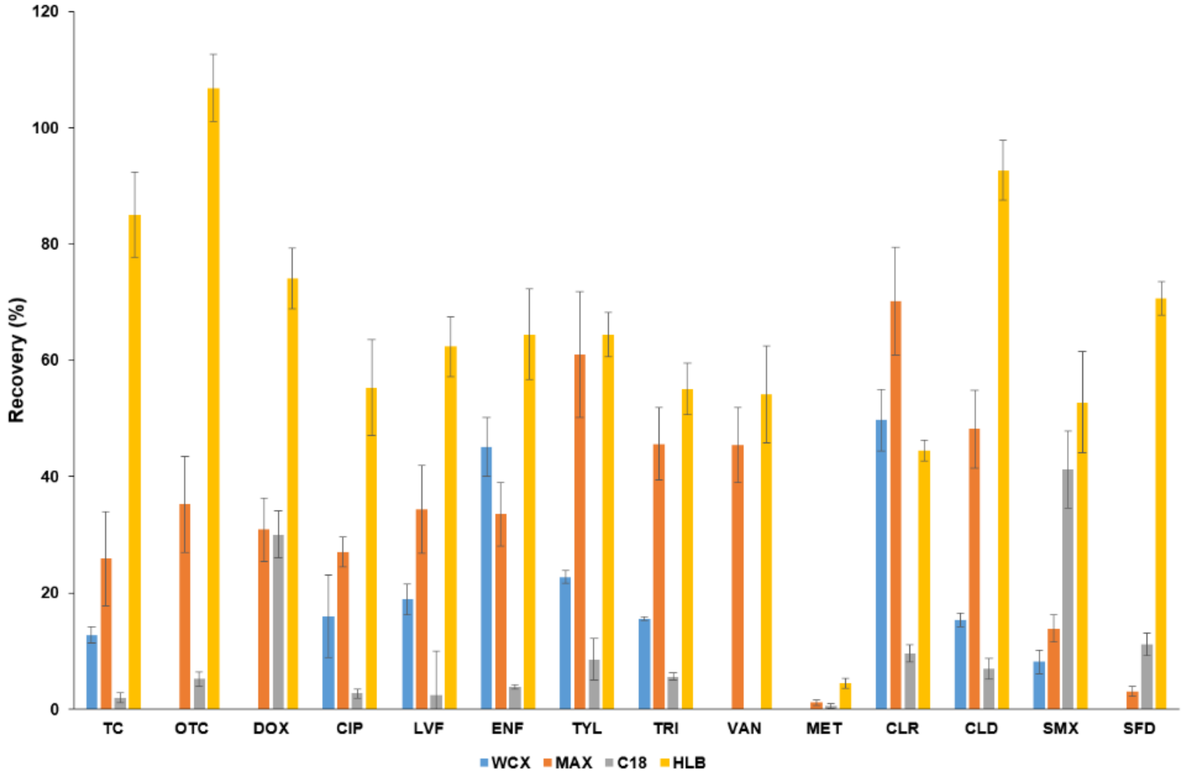

Figure S2. Comparison of sorbents used for purification of extracts in SLE-SPE procedure of parsley leaf. Eluent composition for MAX, C18, HLB: 0.1% AcA in MeOH (10 mL), 0.1% NH_3 in MeOH (10 mL), for WCX: MeOH (3 mL), 2% FA in MeOH/ACN (2:8; *v*/*v*) (3 mL).

Table S1. Variable parameters in tested SLE procedures.

Abbrev.	Solvent	Volume (mL)
SLE L1	MeOH	10
SLE L2	ACN	10
SLE L3	MeOH:ACN (1:1; *v/v*)	10
SLE L4	McIlvaine buffer (pH = 4)	10
SLE L5	McIlvaine buffer (pH = 4):ACN (1:1; *v/v*)	10
SLE L6	McIlvaine buffer (pH = 4):MeOH (1:1; *v/v*)	10
SLE L7	McIlvaine buffer (pH = 4):ACN (1:1; *v/v*)	20 (2 × 10)
SLE L8	0.2 M NaOH:acetone (1:1; *v/v*)	20 (2 × 10)
SLE L9	MeOH:EtOH:McIlvaine buffer (pH = 4) (30:20:45; *v/v/v*)	20
SLE L10	MeOH:EtOH:H_2O (30:20:45; *v/v/v*)	20
SLE L11	MeOH:EtOH:McIlvaine buffer (pH = 4) (30:20:45; *v/v/v*)	30 (15 × 2)

Table S2. Variable parameters in tested SLE-SPE procedures.

Abbrev.	SLE Solvent (*v/v*) (volume (mL))	Sample Volume (mL)	SPE Elution Solvent (volume (mL))
SLE-SPE L1	McIlvaine buffer (pH = 4):ACN (1:1; *v/v*) (10)	300 (2% org.)	MeOH (6)<br>0.1% AcA in MeOH (4)
SLE-SPE L2	McIlvaine buffer (pH = 4):MeOH (1:1; *v/v*) (10)<br>McIlvaine buffer (pH = 4):ACN (1:1; *v/v*) (10)	300 (3% org.)	MeOH (6)<br>0.1% AcA in MeOH (4)
SLE-SPE L3	McIlvaine buffer (pH = 4):MeOH (1:1; *v/v*) (10)<br>McIlvaine buffer (pH = 4):ACN (1:1; *v/v*) (10)	300 (3% org.)	MeOH (10)<br>0.1% AcA in MeOH (10)
SLE-SPE L4	McIlvaine buffer (pH = 4):MeOH (1:1; *v/v*) (10)<br>McIlvaine buffer (pH = 4):ACN (1:1; *v/v*) (10)	300 (3% org.)	0.1% AcA in MeOH (10)<br>0.1% NH_3 in MeOH (10)
SLE-SPE L5	McIlvaine buffer (pH = 4):ACN (1:1; *v/v*) (15)<br>McIlvaine buffer (pH = 4):ACN (1:1; *v/v*) (15)	500 (3% org.)	2% FA in MeOH:ACN (2:8; v/v) (10)<br>0.1% NH_3 in MeOH (10)
SLE-SPE L6	McIlvaine buffer (pH = 4):ACN (1:1; *v/v*) (15)<br>McIlvaine buffer (pH = 4):ACN (1:1; *v/v*) (15)	500 (3% org.)	0.1% AcA in MeOH (10)<br>0.1% NH_3 in MeOH (10)

Table S3. The application of different solvents to SLE procedure for parsley leaf samples.

Solvent	Recovery (%) (SD)													
	**TC**	**OTC**	**DOX**	**CIP**	**LVF**	**ENF**	**TYL**	**TRI**	**VAN**	**MET**	**CLR**	**CLD**	SMX	SFD
SLE L1	30.9 (2.4)	34.4 (2.6)	30.2 (1.7)	34.3 (3.0)	50.4 (6.3)	68.7 (6.4)	68.2 (7.3)	60.9 (5.0)	-	66.0 (4.6)	80.2 (8.4)	53.3 (7.1)	63.8 (5.9)	65.7 (5.6)
SLE L2	2.9 (1.8)	-	-	14.9 (4.8)	28.2 (7.9)	48.4 (9.2)	63.2 (4.3)	15.8 (3.8)	-	52.6 (7.7)	79.2 (7.0)	49.0 (7.3)	53.6 (8.6)	54.4 (9.0)
SLE L3	19.6 (5.2)	19.5 (5.1)	18.8 (4.8)	35.4 (3.4)	38.9 (8.4)	68.8 (6.1)	67.4 (2.8)	63.7 (4.3)	-	65.1 (5.2)	87.5 (4.3)	45.2 (9.9)	62.3 (7.4)	67.3 (7.5)
SLE L4	78.1 (4.9)	90.2 (5.5)	72.2 (5.4)	73.3 (3.1)	31.5 (5.1)	61.4 (2.6)	73.6 (2.7)	65.6 (2.1)	102.8 (4.3)	82.7 (4.1)	76.8 (2.9)	39.0 (5.5)	63.2 (2.5)	86.4 (4.4)
SLE L5	77.6 (5.0)	91.6 (5.7)	71.3 (5.3)	87.7 (4.3)	40.1 (12.4)	85.5 (5.2)	73.8 (3.9)	74.5 (3.8)	77.8 (8.3)	78.7 (2.7)	85.2 (5.6)	42.9 (6.8)	76.8 (2.4)	95.9 (4.3)
SLE L6	66.5 (5.0)	79.6 (2.0)	64.2 (5.9)	99.3 (2.8)	30.9 (5.5)	100.0 (4.2)	92.7 (2.7)	90.4 (2.7)	65.2 (3.8)	88.8 (1.0)	97.0 (4.0)	41.2 (3.9)	66.2 (2.5)	90.4 (2.8)
SLE L7	93.2 (5.1)	96.6 (1.7)	73.4 (0.4)	66.0 (5.8)	70.3 (3.6)	68.5 (3.9)	89.9 (4.8)	84.3 (2.9)	78.8 (16.0)	91.4 (4.1)	96.7 (5.2)	96.1 (4.2)	89.0 (5.2)	89.7 (1.0)
SLE L8	26.1 (2.3)	35.8 (3.1)	21.3 (3.4)	35.6 (5.4)	30.3 (3.4)	40.1 (3.6)	3.5 (0.3)	89.0 (3.2)	87.4 (6.6)	43.7 (1.3)	56.9 (9.2)	65.5 (6.7)	69.2 (4.9)	60.6 (4.6)
SLE L9	60.7 (4.4)	62.6 (2.7)	65.1 (2.5)	52.7 (3.4)	48.3 (2.3)	50.8 (2.0)	62.9 (2.8)	71.7 (1.5)	51.60 (2.7)	80.4 (2.0)	57.8 (2.2)	65.4 (3.8)	60.3 (2.1)	87.5 (1.1)
SLE L10	37.6 (1.8)	38.0 (1.4)	36.1 (0.9)	34.4 (2.5)	35.9 (1.3)	38.6 (2.2)	61.3 (5.2)	68.5 (3.3)	46.1 (3.2)	86.3 (5.3)	55.6 (3.8)	65.6 (2.5)	56.2 (4.2)	87.3 (5.0)
SLE L11	69.4 (3.5)	73.0 (1.9)	66.6 (2.0)	62.6 (4.5)	57.3 (2.8)	58.1 (2.0)	71.6 (2.3)	77.4 (3.1)	61.9 (1.7)	87.3 (2.2)	68.7 (3.7)	73.5 (2.5)	70.6 (2.3)	98.7 (3.8)

** The best SLE conditions are highlighted in color*

molecules

MDPI

Table S4. The analytical method parameters and extraction recovery of antibiotics in parsley root samples at three different concentrations (n = 6).

Analyte	Linear Range (ng·g⁻¹)	$R^{2,a}$	LOD[b] (ng·g⁻¹)	LOQ[c] (ng·g⁻¹)	Concentration (ng·g⁻¹)	Precision *CV(%)[d]*	Accuracy *RE(%)[e]*	ME[f] (%)	Recovery ± SD (%)
**TC**	5 – 1200	0.9930	1.6	5.0	100	5.10	7.56	−4.69	70.0 ± 3.7
					400	5.87	5.64		72.2 ± 3.8
					1000	5.58	5.05		81.8 ± 8.3
**OTC**	1 – 1200	0.9958	0.3	1.0	100	5.93	5.97	3.45	80.9 ± 5.6
					400	5.78	5.42		80.7 ± 2.6
					1000	5.77	2.33		90.6 ± 1.6
**DOX**	1 – 1200	0.9988	0.3	1.0	100	9.60	4.71	7.81	68.8 ± 0.5
					400	5.70	4.12		70.1 ± 3.1
					1000	2.57	4.04		86.3 ± 13.7
**SMX**	1 – 1200	0.9953	0.3	1.0	100	4.30	2.23	−4.06	45.1 ± 6.9
					400	4.87	1.78		45.7 ± 1.1
					1000	4.53	1.35		54.2 ± 4.9
**SFD**	1 – 1200	0.9968	0.3	1.0	100	4.05	4.55	−11.35	46.5 ± 6.1
					400	6.62	6.23		48.5 ± 2.3
					1000	6.54	0.47		52.9 ± 5.4
**CIP**	1 – 1200	0.9858	0.3	1.0	100	7.76	2.89	0.49	84.0 ± 1.9
					400	6.42	0.73		85.8 ± 7.8
					1000	7.10	2.53		94.4 ± 6.9
**LVF**	1 – 1200	0.9967	0.3	1.0	100	4.86	3.49	−0.07	72.0 ± 3.9
					400	3.76	2.64		75.6 ± 5.0
					1000	3.97	1.67		80.5 ± 11.8
**ENF**	1 – 1200	0.9988	0.3	1.0	100	5.91	2.91	-3.94	72.6 ± 3.1
					400	5.84	3.92		76.6 ± 5.5
					1000	5.38	4.37		87.1 ± 7.4
**MET**	1 – 1200	0.9969	0.3	1.0	100	7.26	3.14	−2.72	50.6 ± 2.8
					400	7.83	4.28		51.2 ± 1.2
					1000	7.12	0.40		51.7 ± 2.1
**TRI**	1 – 1200	0.9970	0.3	1.0	100	7.04	3.64	−5.38	77.6 ± 5.0
					400	7.58	3.29		82.5 ± 3.0
					1000	5.96	1.14		89.9 ± 3.4
**VAN**	1 – 1200	0.9896	1.6	5.0	100	5.06	1.99	−7.47	78.2 ± 2.9
					400	5.87	1.80		80.5 ± 2.4
					1000	8.74	1.20		90.7 ± 10.4
**TYL**	1 – 1200	0.9925	0.3	1.0	100	6.77	5.92	0.35	73.1 ± 4.8
					400	3.95	4.78		74.7 ± 7.7
					1000	3.23	1.31		80.7 ± 7.3
**CLR**	1 – 1200	0.9971	0.3	1.0	100	7.53	3.11	−6.31	79.7±3.2
					400	5.67	2.78		81.1 ± 3.7
					1000	4.70	2.41		95.6 ± 2.2
**CLD**	1 – 1200	0.9962	0.3	1.0	100	6.80	6.49	11.31	57.3 ± 2.6
					400	7.72	6.00		57.8 ± 3.4
					1000	5.83	6.12		56.7 ± 5.5

[a]R^2: coefficient of determination; [b]LOD: limit of detection; [c]LOQ: limit of quantification; [d]CV: coefficient of variation; [e]RE: relative error; [f]ME: matrix effects

Table S5. The analytical method parameters and extraction recovery of antibiotics in parsley leaf samples at three different concentrations (n = 6).

Analyte	*Linear Range (ng·g⁻¹)*	$R^{2,a}$	*LOD[b] (ng·g⁻¹)*	*LOQ[c] (ng·g⁻¹)*	Concentration (ng·g⁻¹)	Precision CV(%)[d]	Accuracy RE(%)[e]	ME[f] (%)	Recovery ± SD (%)
**TC**	5 – 1200	0.9930	1.6	5.0	100	4.37	3.03	7.96	60.3 ± 8.7
					400	3.72	3.01		65.2 ± 1.15
					1000	3.56	2.96		83.2 ± 4.8
**OTC**	1 – 1200	0.9958	0.3	1.0	100	7.90	2.45	4.61	70.1 ± 12.9
					400	6.07	1.79		79.0 ± 4.2
					1000	4.69	1.68		97.1 ± 5.9
**DOX**	1 – 1200	0.9988	0.3	1.0	100	3.56	8.31	4.38	80.7 ± 3.5
					400	3.22	4.38		80.1 ± 6.0
					1000	2.39	3.20		89.6 ± 3.4
**SMX**	1 – 1200	0.9953	0.3	1.0	100	4.19	7.14	1.64	70.2 ± 1.9
					400	3.69	4.40		71.5 ± 1.5
					1000	2.88	3.57		77.8 ± 4.5
**SFD**	1 – 1200	0.9968	0.3	1.0	100	3.79	6.51	2.35	55.6 ± 3.9
					400	3.51	6.64		55.4 ± 4.3
					1000	2.53	5.19		81.3 ± 7.4
**CIP**	1 – 1200	0.9858	0.3	1.0	100	4.28	5.15	5.95	60.3 ± 2.1
					400	1.83	3.23		62.0 ± 3.7
					1000	1.25	1.61		67.3 ± 3.7
**LVF**	1 – 1200	0.9967	0.3	1.0	100	5.00	9.52	6.88	59.7 ± 6.0
					400	4.89	4.45		61.3 ± 2.1
					1000.	4.68	2.57		74.9 ± 4.6
**ENF**	1 – 1200	0.9988	0.3	1.0	100	4.76	11.42	−6.74	60.1 ± 3.6
					400	2.63	4.81		61.1 ± 1.2
					1000	1.98	3.52		88.3 ± 5.7
**MET**	1 – 1200	0.9969	0.3	1.0	100	5.62	6.07	−1.07	84.4 ± 7.8
					400	5.51	4.50		88.9 ± 9.7
					1000	4.81	1.90		85.3 ± 11.3
**TRI**	1 – 1200	0.9970	0.3	1.0	100	5.67	3.81	6.50	56.6 ± 3.5
					400	5.18	1.70		67.9 ± 3.6
					1000	4.64	0.49		80.5 ± 7.7
**VAN**	5 – 1200	0.9896	1.6	5.0	100	6.82	2.56	2.17	46.0 ± 1.9
					400	6.74	2.48		51.4 ± 4.1
					1000	6.21	2.42		68.1 ± 8.37
**TYL**	1 – 1200	0.9925	0.3	1.0	100	6.11	7.40	−4.84	85.4 ± 12.1
					400	4.90	4.82		83.1 ± 3.8

					1000	2.09	2.99		89.9 ± 3.0
**CLR**	1 – 1200	0.9971	0.3	1.0	100	5.94	7.07	2.26	64.3 ± 1.9
					400	5.20	6.64		65.4 ± 9.4
					1000	4.90	5.28		61.9 ± 5.9
**CLD**	1 – 1200	0.9962	0.3	1.0	100	3.30	6.47	−1.49	65.3 ± 5.4
					400	2.37	6.30		66.5 ± 3.3
					1000	1.81	0.72		80.8 ± 2.8

[a]R^2: coefficient of determination; [b]LOD: limit of detection; [c]LOQ: limit of quantification; [d]CV: coefficient of variation; [e]RE: relative error; [f]ME: matrix effects
